# Western Mode of Accosting Physicians

**Published:** 1850-01

**Authors:** 


*Western mode of accosting Physicians.*—A correspondent of the Western Journal of Medicine and Surgery, residing in Jamestown, Ohio, states that the universal custom of the people in his neighborhood, when they address a physician, is to call him "*Doc;*" and that in speaking of the attendance of a medical man, the usual phrase is "*waiting on*" the patient. A common form of expression, when any one wishes to enquire about the sick, is, "*Doc,* how is that patient you are *waiting* on?"

